# Correlative Nanoscale 3D Imaging of Structure and Composition in Extended Objects

**DOI:** 10.1371/journal.pone.0050124

**Published:** 2012-11-19

**Authors:** Feng Xu, Lukas Helfen, Heikki Suhonen, Dan Elgrabli, Sam Bayat, Péter Reischig, Tilo Baumbach, Peter Cloetens

**Affiliations:** 1 Institute for Synchrotron Radiation, Karlsruhe Institute of Technology, Eggenstein-Leopoldshafen, Germany; 2 European Synchrotron Radiation Facility, Grenoble, France; 3 Institut National de l'Environnement Industriel et des Risques, Verneuil en Halatte, France; 4 Centre Hospitalier Universitaire Amiens, Université de Picardie Jules Verne, Amiens, France; Illinois Institute of Technology, United States of America

## Abstract

Structure and composition at the nanoscale determine the behavior of biological systems and engineered materials. The drive to understand and control this behavior has placed strong demands on developing methods for high resolution imaging. In general, the improvement of three-dimensional (3D) resolution is accomplished by tightening constraints: reduced manageable specimen sizes, decreasing analyzable volumes, degrading contrasts, and increasing sample preparation efforts. Aiming to overcome these limitations, we present a non-destructive and multiple-contrast imaging technique, using principles of X-ray laminography, thus generalizing tomography towards laterally extended objects. We retain advantages that are usually restricted to 2D microscopic imaging, such as scanning of large areas and subsequent zooming-in towards a region of interest at the highest possible resolution. Our technique permits correlating the 3D structure and the elemental distribution yielding a high sensitivity to variations of the electron density via coherent imaging and to local trace element quantification through X-ray fluorescence. We demonstrate the method by imaging a lithographic nanostructure and an aluminum alloy. Analyzing a biological system, we visualize in lung tissue the subcellular response to toxic stress after exposure to nanotubes. We show that most of the nanotubes are trapped inside alveolar macrophages, while a small portion of the nanotubes has crossed the barrier to the cellular space of the alveolar wall. In general, our method is non-destructive and can be combined with different sample environmental or loading conditions. We therefore anticipate that correlative X-ray nano-laminography will enable a variety of *in situ* and *in operando* 3D studies.

## Introduction

Progress in key technologies is increasingly related to the ability of tailoring material properties [1]–[4]. To this end, characterizing material structure and composition on various length scales, from the engineering level down to the nanoscale, is of fundamental interest. In modern life sciences, understanding processes and functions at the cellular, sub- and intercellular levels is vital [5], [6]. In particular, for the study of trace metals in biological systems [7], imaging methods that are sensitive both to elements and the morphology are essential.

Therefore, a broad ensemble of two-dimensional (2D) microscopy and three-dimensional (3D) tomography methods has been developed for micro and nanoscale imaging in the last two decades, based on ultrasound, electron, ion, X-ray and atom probe techniques [8]–[10]. Here the choice of the best-suited technique to tackle a particular imaging problem is not so much a sole question of the achievable resolution but rather one of an optimum compromise between sufficient contrast, exposure time, well adapted resolution, reasonable field of view (FOV), and feasibility of sample preparation. Information in three dimensions can be obtained destructively by extending 2D surface scanning techniques in the third direction, for example via serial sectioning or ion beam milling. Alternatively it can be obtained non-destructively via confocal scanning or tomographic projection techniques.

In 2D optical microscopy, a very important property is the ability to study the specimen at large field of view (FOV), and then choosing an interesting area for closer inspection with higher magnification. In X-ray imaging the achievable FOV is confined by the limited scanning time or number of detector elements. For example hard X-ray computed tomography (CT) has recently entered a resolution range of 50 nm pixel size, giving a FOV of only 50 to 100 µm. For good quality CT imaging, the specimen cannot be larger than the FOV. Therefore a suitable region of interest (ROI) has to be identified and physically extracted from the specimen before imaging. Such destructive intervention requires prior knowledge about the location of the features of interest and is not nearly as flexible as the ability to choose the ROI online as in 2D microscopy. Furthermore, the physical extraction of the ROI also seriously increases the risk of unintended modifications of the micro and nanostructures in the ROI. Additionally, in some cases such as functionality studies or successive investigations, preserving the specimen’s integrity is a prerequisite. Consequently, with increasing spatial resolution and proportionally decreasing size of the FOV, the ability to image intact specimens is essential.

The above challenge can be overcome by applying principles of laminography. Synchrotron computed laminography (CL) [11], [12] is a generalization of synchrotron tomography [13] where the tomographic rotation axis can be tilted with respect to the beam direction by an angle smaller than 90°. This additional degree of freedom makes laminography flexible to image specimens that are extended in two dimensions [14] (but comparable to the field of view in the 3^rd^ dimension), enabling 3D imaging of intact and large specimens. We gain several benefits: 1) high resolution 3D imaging becomes possible within laterally large specimen, presently up to a few square centimeters, in analogy to standard 2D light microscopes by zooming into selected ROIs with suitably high resolution, 2) in this way, multi-scale 3D imaging, e.g., of hierarchical structures, becomes possible, 3) *in situ* nanoscale imaging of micro devices during operation and testing cycles becomes possible. In biology the use of flat samples allows standard sample preparation techniques, such as cutting a slice out of the sample or growing cells on an appropriate substrate, to be employed. Such samples are generally used in 2D microscopy as well as in 2D X-ray fluorescence imaging. In this context, laminography offers the possibility to study the same samples, but with the ability to gain three-dimensional information. Using synchrotron radiation, CL has been combined so far with absorption [12], phase [15] and fluorescence contrast [16] providing spatial resolutions down to a few µm.

## Results

**Figure 1:**
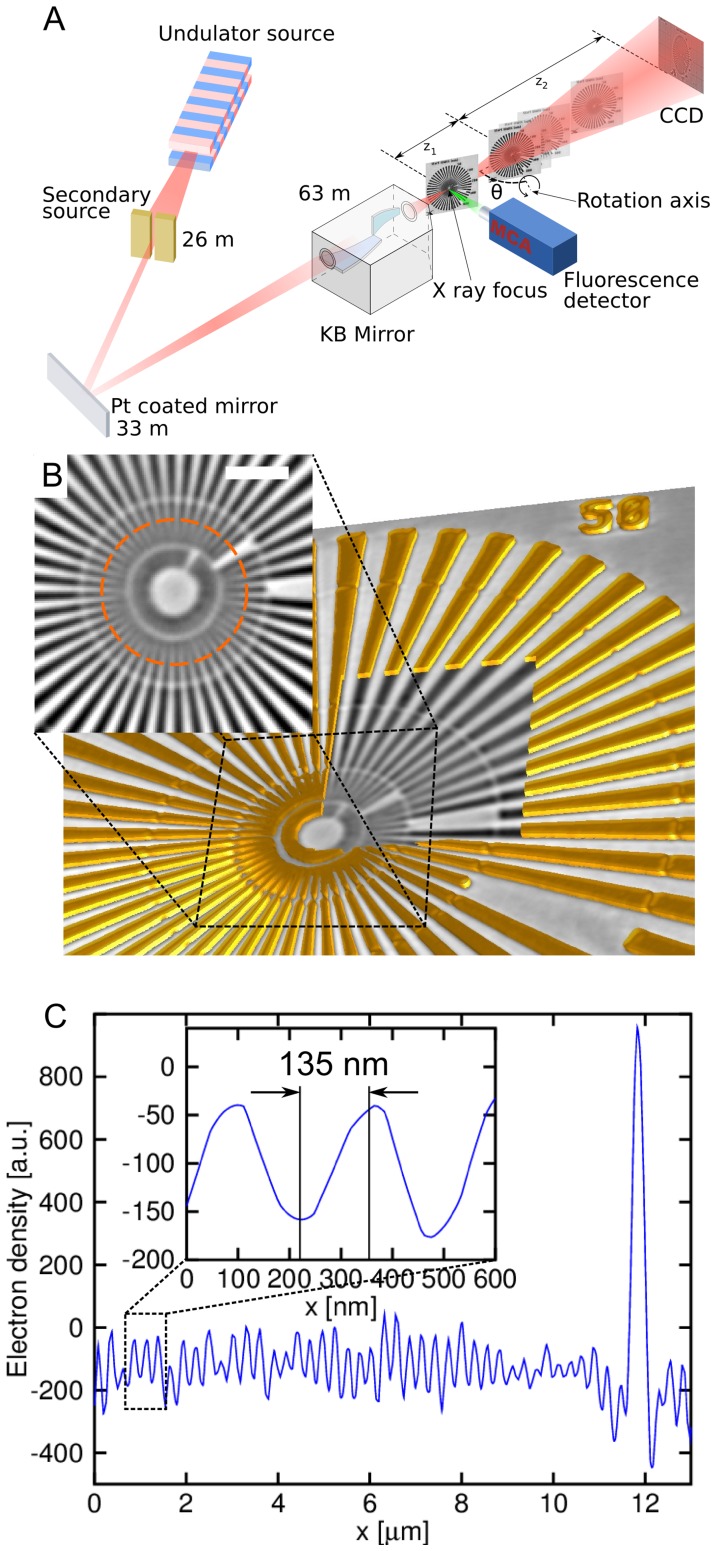
Principle of correlative nanolaminography. (A) Schematic of the nanolaminography setup on ID22NI at the ESRF. The beam is focused by multilayer coated Kirkpatrick-Baez optics. The specimen is placed in the focus to raster scan the fluorescence spectra. The specimen is placed downstream of the focus to obtain a full-field magnified holographic image on the 2D detector. The tomographic rotation axis is inclined by the laminographic angle θ with respect to the beam direction. (B) 3D rendering of a lithographically fabricated Siemens star test pattern: the window reveals a single slice from the reconstructed 3D volume and the inset shows the center of the test pattern (scale bar 2 µm). (C) Profile plot along the circle shown in panel b. The inset shows a 135 nm half period achieved as lateral resolution in the 3D image.

We implemented a high-precision specimen stage for laminography at the European Synchrotron Radiation Facility (ESRF) nano-imaging endstation ID22NI (Figure 1A). The setup permits obtaining 3D structural and elemental contrast within the same setup. The size of the focal spot and the precision of the sample scanning allow us to gain an order of magnitude in spatial resolution over previous laminography approaches. Briefly, a single-line undulator source and subsequent mirror-based focusing optics [17] produce a monochromatic X-ray focus of about 47×82 nm^2^ with a flux of more than 10^12^ photons/s.

The mechanical setup contains a horizontal tomographic rotation axis which can be tilted in the horizontal plane towards the beam direction in order to set the optimum laminographic angle (Figure 1A). Projection microscopy provides propagation based phase contrast with cone beam geometrical magnification leading to spatial resolutions much better than the detector pixel size. Full-field intensity measurements at four focus-specimen distances allow one to perform ultrasensitive phase retrieval [18], [19]. The fluorescence signal is recorded while raster-scanning the specimen in the focal plane, giving the spatial distribution of elements with atomic numbers above 13, down to ppm sensitivity. The setup enables full-field phase laminography in combination with scanning fluorescence laminography. The former is typically carried out at effective pixel size of 50 nm, and the latter at pixel size chosen between 50 nm and 500 nm, depending on the size of the required region of interest and the available scanning time. We examine a lithographically fabricated nanostructure to give experimental evidence for the resolution in the 3D reconstructed phase image. The structure contains a Siemens star pattern made of a 200 nm (+/−10%) thick gold layer, supported by a 300 nm thick Si_3_N_4_ membrane. The reconstructed slices (Figure 1B) and the profile plot in Figure 1C confirm that the spatial resolution for full field imaging is better than 135 nm in the specimen plane. To achieve this result, a small focal spot size, high mechanical precision of the specimen rotation, and good long-term mechanical stability are essential. The through-plane resolution is generally worse in laminography, and the maximum spatial frequencies along the rotation axis direction are limited by a factor of cos(*θ*) – where *θ* is the axis inclination angle according to Fig. 1– compared to the case of tomography. It is more difficult to quantify, however, as the artefacts introduced in this direction depend on the object dimensions in all three directions [14].

**Figure 2:**
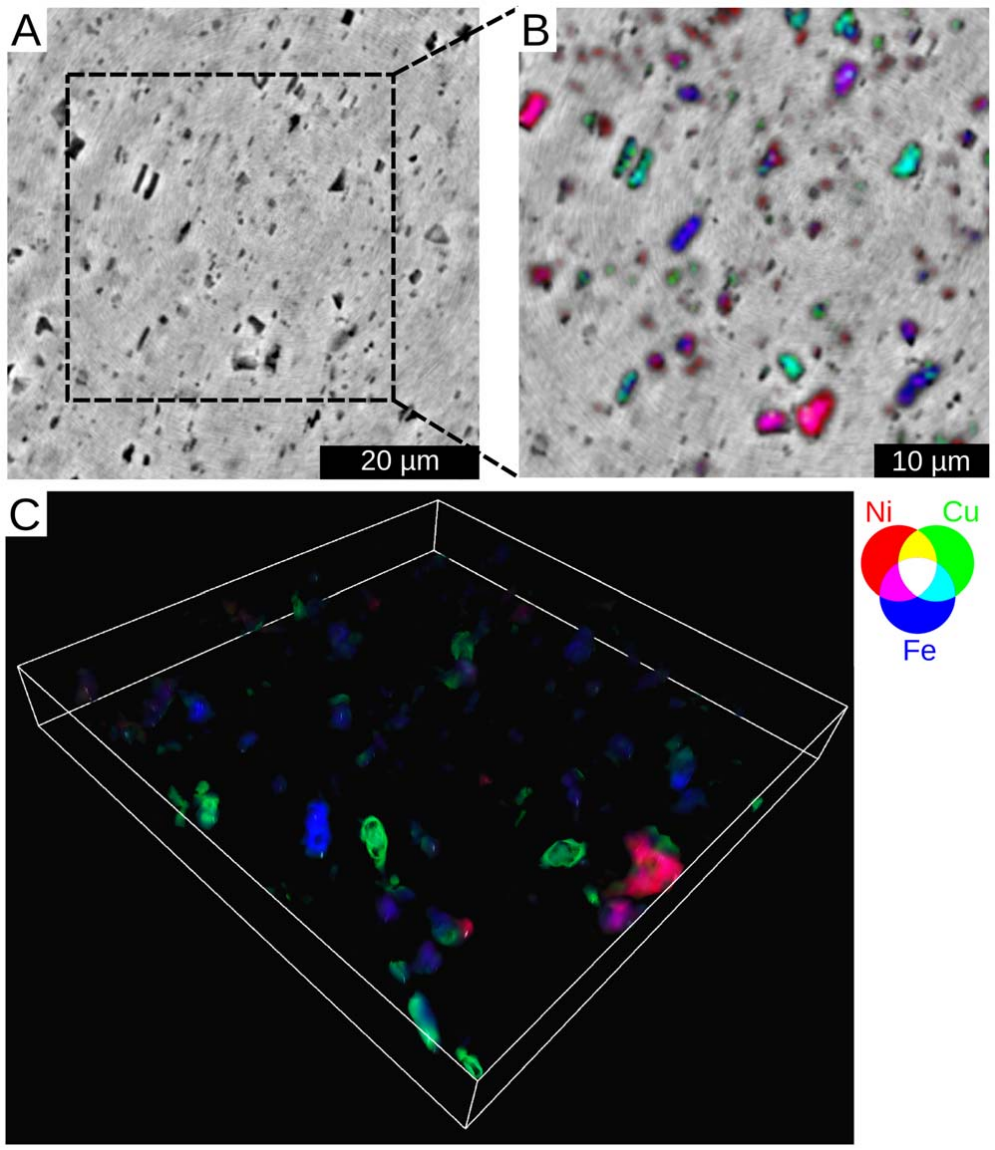
Correlative nanolaminography for materials research. A 2D slice of the structure and the elemental distribution in an aluminum foil, taken at 3 µm below the surface. (A) Structure obtained by full field phase laminography. The denser second-phase particles are dark. (B) Co-localization of Ni, Cu and Fe overlaid with the structure. (C) Co-localization of Ni, Cu and Fe shown in three dimensions.

The way nanolaminography permits materials diagnostics is illustrated in terms of 3D correlative imaging of the electron density and major chemical elements in a 42 micron thick AA8079 aluminum foil. Fig. 2 depicts the correspondence between 3D phase and fluorescence images. By reconstructing the 3D electron density we detect plenty of particles denser than the surrounding Al matrix. These second-phase particles that were of similar density in the phase contrast images are revealed to be of different composition in the fluorescence 3D images. They are mainly composed of Fe, Ni and Cu, with varying content of elements in the different particles. Ni and Cu were found to be inside particles always containing Fe, but never occurring together. The discovery of multiple particles with Ni was especially interesting, because Ni is not an alloying element of the alloy AA8079. Generally this example demonstrates that by overlaying the laminographically reconstructed 3D structural and multi-elemental distributions, intermetallic phases, impurities, voids, and cracks are locatable and can be identified without specimen destruction. This knowledge is very important for optimizing the material properties of engineering alloys.

In biology, imaging trace element distributions along with the structure is highly important. The study of endogenous distributions of trace elements offers insight into pathologic conditions, such as cancer and neurodegenerative diseases [20], [21], while using antibodies tagged with heavy elements allows one to study specific intracellular targets such as receptors [22]. Localization of distributions of endogenous heavy elements inside cell and tissue structures enables further understanding of toxic effects of environmental pollutants.

Nanoparticles in particular have become a major concern as environmental pollutants because their use in the industry has increased. With their high surface–to–volume ratio, high mobility, and easy penetration through membranes in biological systems, nanoparticles could have strong toxic effects even when the elemental composition of the particle is harmless to the organism. To understand the toxic effects of nanoparticles, analyzing their pathways inside tissues and cells is important. Airborne nanoparticles can easily penetrate deep into the lung; especially carbon nanotubes (CNT) have been shown to have highly toxic effects at the alveolar level [23], [24]. Electron microscopy studies have been used to localize CNTs inside cellular structures, but the FOV of such studies is limited [23]. Nanolaminography offers a complementary, multi-scale approach to examine large tissue specimens.

**Figure 3:**
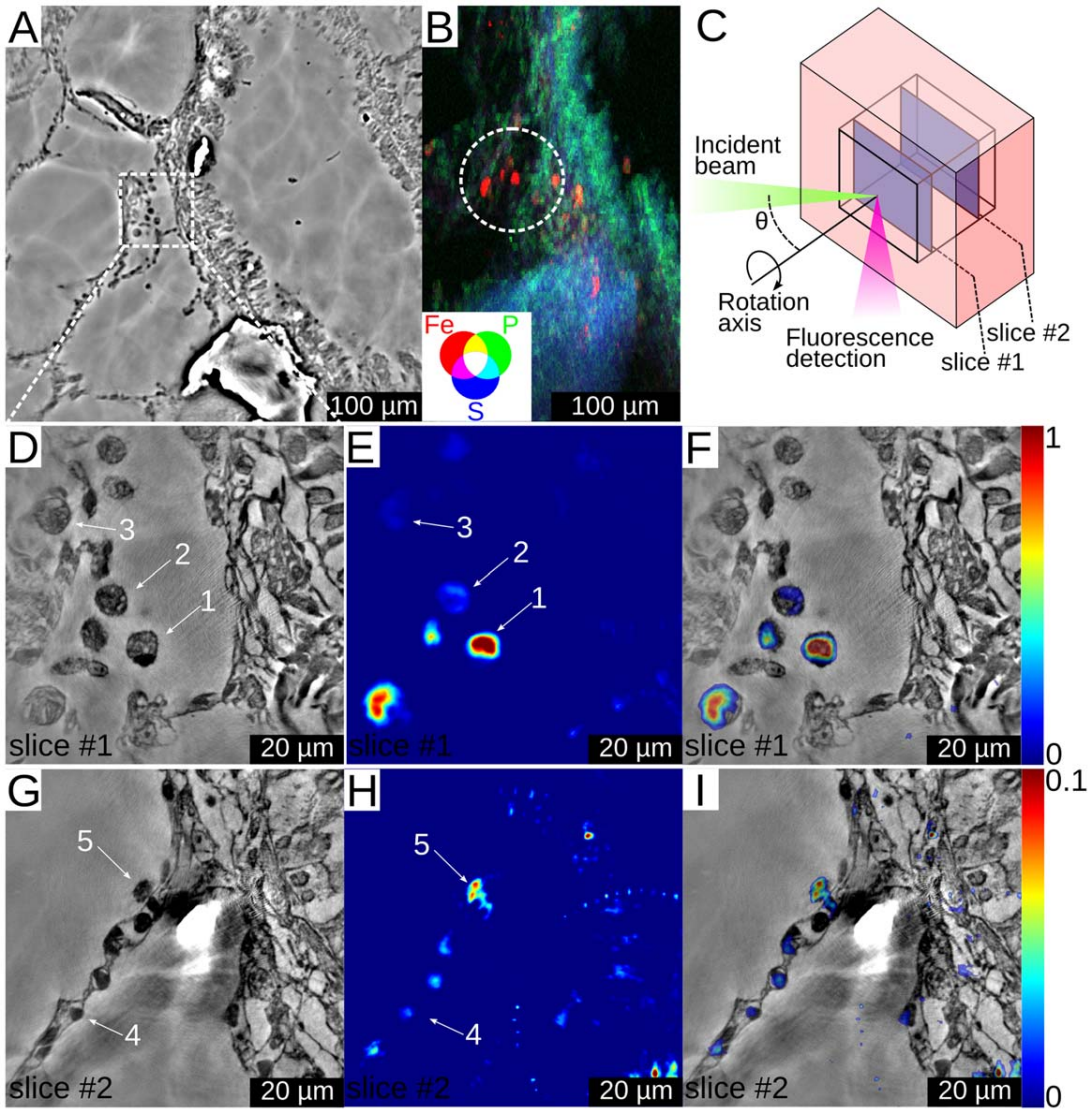
Imaging of carbon nanotube contamination in lung tissue. (A) Slice from a low resolution electron density volume. (B) Low resolution 2D fluorescence image of the specimen showing iron, phosphorus and sulfur distributions. The circle indicates the ROI that was chosen for higher resolution imaging. The scanned area was about 0.2×0.3 mm^2^ and the step size was 1.8 µm. (C) A schematic representation of the imaging geometry, showing the ROI inside the sample and the two slices that were chosen for further analysis. Phase contrast slice with 60 nm pixel size (D), Fe fluorescence slice with 500 nm pixel size (E) and correlative image of Fe and phase contrast (F) for slice #1 in the lung specimen. Numbers 1, 2, and 3 indicate positions of alveolar macrophages. Phase contrast (G), Fe fluorescence signal (H) and correlative image of Fe and phase contrast (I) for slice #2 in the lung specimen. Number 4 indicates the position of a type 1 pneumocyte, while number 5 indicates the position of an alveolar macrophage. Notice the factor of 10 difference in the relative fluorescence signal between parts (E, F) and (H, I).

We have studied the biodistribution of single-walled carbon nanotubes (SWCNT) that were instilled in rat lungs. A well-established animal preparation protocol [24] was used for preparing lung samples. Since the nanotubes contained iron impurities (∼10.1% mass) the detection of excess iron by fluorescence imaging indicates agglomerations of nanotubes and their localization in the tissues. The main results are shown in Figure 3. A lung specimen was first screened by low resolution phase contrast imaging (Figure 3A) and a coarse resolution 2D fluorescence scan (Figure 3B). Fluorescence hotspots indicate CNT agglomerations in some cellular structures, and we zoomed into one of these areas for laminographic high resolution imaging.

Two slices at different depths were chosen from the reconstructed 3D data for closer inspection (see Figure 3C). The electron density (Figure 3D), the fluorescence image (Figure 3E), and the correlated image (Figure 3F) show that in slice #1 there is an excess of iron inside specific cellular structures (Video S1). By careful examination of the electron density images, the cells in question can be identified as alveolar macrophages (AM), clearly indicated by their size (about 12 µm), location (close to the alveolar surface), and the shapes of their nuclei (single, round). The excess content of iron inside some of the AMs indicates the presence of nanotubes. This confirms the finding from electron microscopy that AMs are able to phagocyte nanotubes from the lungs [23].

Figures 3G, 3H and 3I correspond to slice #2 where one part of an alveolar wall structure is depicted. They show hotspots of iron inside cellular structures of the wall. From their shape, the cells can be identified as type-1 pneumocytes and one AM. The iron concentration in the pneumocytes is lower than that in the AMs of slice #1 by more than a factor of 10, but substantially higher than in the surrounding tissue. Although the iron could be endogenous, comparison of the different cells in the lung wall shows that some have much higher quantities of iron than others. The images suggest–for the first time in an *in vivo* experiment–that nanotubes have entered in small amounts inside some of the pneumocytes.

## Discussion

Nanolaminography allowed focusing on a region of the lung that was found to be highly different from the surrounding tissue. This multi-scale approach is beneficial to biology and other applications where the specimen’s heterogeneity makes a single ROI unrepresentative of the entire specimen, and where the interesting regions may be far apart from each other. Correlative images allowed establishing correspondence between trace-metal excess content and the hosting cell types.

In summary, we have developed a technique for correlative 3D imaging of both structure and elemental composition of extended objects. The geometry is ideal to extend 2D X-ray fluorescence microscopy to 3D investigations. Due to the small size of the x-ray focal spot and the mechanically precise sample handling, our setup presently achieves a resolution of about 100 nm. It seems only a matter of time that higher spatial resolutions below 10 nm will be achieved via further reduction of the focused beam size [25] or in combination with coherent diffraction imaging [26]. Developments in faster data collection for fluorescence scans [27] will make high resolution fluorescence imaging of larger ROIs more practical. Our contact-free and non-destructive imaging method is particularly suited for *in situ* and *in operando* studies in materials research and technology, and in life sciences.

## Materials and Methods

### Ethics Statement

The experimental protocol involving the rats has been approved by the INERIS (Institute National de l'Environnement Industriel et des Risques, Verneuil-en-Halatte, France) local ethical committee for animals in research.

### Sample Preparation

Rats were anesthetized and were intratracheally instilled with SWCNT dispersed with bovine serum albumin (BSA) as previously described [24]. Seven days later the rats were sacrificed. Tissue specimens from the lungs were fixed in 10% formaldehyde and processed routinely for embedding in paraffin. Slices of 40 µm thickness and 3×3 mm^2^ cross section were cut from the paraffin blocks and were used for 3D imaging.

### Experiment

Nanoscale electron density and elemental composition laminography was implemented at the nano-imaging endstation ID22NI of the European Synchrotron Radiation Facility (ESRF). A Kirkpatrick-Baez mirror system was used to focus X-rays of 17 keV energy to a focal spot of 47 (V) ×82 (H) nm^2^ (FWHM) with a flux of more than 10^12^ photons/s in the focus. To get laminographic images, we set the sample rotation axis in a 60° inclination with respect to the incoming beam. For electron density imaging, the X-ray focus was used as a virtual source, and magnified images of the sample (at 60 nm pixel size) were obtained with X-ray projection microscopy. Phase retrieval was then performed on the images with the contrast transfer function method [19] to get the X-ray phase modulation due to the sample. The lateral field of view was 90×90 µm^2^ with the thickness of the sample that could be imaged being approximately equal to the lateral field of view. The elemental composition was imaged with the sample at the X-ray focus. An energy sensitive detector, placed at 90 degrees from the incident beam direction, was used for detecting the characteristic fluorescence photons. The horizontal rotation axis ensured a minimized path of the emitted photons inside the specimen, reducing self-absorption effects. Scans of 100×100 points with a step size of 0.5 µm were acquired for 64 rotation angles. Considering the limited number of recorded projections, an algebraic reconstruction technique [28] was used for 3D reconstruction of the fluorescence data. The implementation used is compatible with laminography in particular because it allows one to determine arbitrary ray geometries. The algorithm used was SART with 5 to 21 iterations. An alternative technique, equally sloped tomography [29], [30], will be investigated in the future as a way to provide good quality reconstructions from a small number of projections. In order to correlate the two types of images the two data sets have to be aligned. The sample remained fixed with respect to the rotation axis for the two scans. Therefore the only uncertainty is in the translation along the rotation axis. This remaining one-dimensional misalignment was less than 1 µm due to the high accuracy of the mechanical setup, and in this experiment this was corrected manually. For more details, please see Supporting Information S1 available on-line.

## Supporting Information

Supporting Information S1 Further details about materials and methods.
(DOC)

Video S1 The movie combines information from three different scans of the lung sample. First, a low resolution electron density volume is shown with 270 nm voxel size. The removal of the low resolution volume reveals a higher resolution electron density volume with 60 nm voxel size. Overlaid with the electron density map is the map of iron concentration in the sample (shown in color).
(MPG)
