# Supplementary material for: Correlative Nanoscale 3D Imaging of Structure and Composition in Extended Objects
Source: PLoS One. 2012 Nov 19;7(11):e50124. doi: 10.1371/journal.pone.0050124 (PMC3501479; doi:10.1371/journal.pone.0050124)
Supplement: Supporting Information S1 — Further details about materials and methods. (DOC) [file pone.0050124.s001.doc]

**Supporting Information S1**

After the discovery by W. C. Röntgen in 1895, X-rays were immediately used for projection radiography. Laminography is an X-ray technique developed in the 1930s by the dutch researcher B. G. Ziedses des Plantes allowing cross-sectional imaging of patients [1]. This concept allowed for the first time to gain (yet blurred) two dimensional (2D) slices of internal structures in laterally extended three dimensional (3D) objects. That was some forty years before 2D computed tomography was developed by Cormack and Hounsfield for medical applications. In the nineties the dramatic progress of powerful computation technology, mathematical algorithms and 2D detector technology led to 3D computed tomography and computed laminography techniques, today being used for manifold applications in medical and industrial imaging. Both techniques have been later successfully established at third generation synchrotron sources taking benefit from their outstanding beam properties [2, 3].

Nanoscale electron density and elemental composition laminography was implemented at the nanoimaging endstation ID22NI of the European Synchrotron Radiation Facility (ESRF). Radiation is generated by a single mode undulator source, and a horizontal slit with 25 µm gap is used to create a secondary source in the horizontal direction. A horizontally reflecting flat mirror cuts higher energies from the beam, and the beam then enters the experimental hutch where the focusing optics are placed. A dynamically bent, multilayer coated Kirkpatrick-Baez mirror system is used for focusing X-rays in the energy range from 17 keV to 29 keV. Presently a focus size of 47 (V) × 82 (H) nm^2^ has been achieved in routine user mode, giving a flux of more than 10^12^ photons/s in the focus with medium monochromaticity (∆*E/E* = 1.5%). This flux corresponds to a photon density of more than 10^8^ photons/nm^2^/s. In our experiments, an X-ray energy of 17.5 keV was used.

The sample stage has linear translation motors in three directions, and additionally rotation for tomographic scanning of the sample. A piezo actuator in one dimension allows for rapid and stable raster scanning of the sample for the fluorescence experiments. The sample resides on a disc that is magnetically attached to the rotation stage, and can be slid with respect to the rotation center to bring different parts of the sample into the field of view. Unlike usual laminography or CT setups where the rotation axis is vertical, the rotation axis in our setup was set to the horizontal plane to optimize 3D fluorescence imaging. To get laminographic images, we set the sample rotation axis in a 60° inclination with respect to the incoming beam.

For electron density imaging, the X-ray focus was used as a virtual source, and magnified images of the sample were obtained with X-ray projection microscopy. X-ray wave propagation in the projection microscopy geometry leads to phase contrast in the images. The high resolution causes the propagation effects to be very strong in the images, and the radiographs before phase retrieval resemble the sample only remotely. However, the phase retrieval methods developed for the parallel beam case work remarkably well. In order to do propagation based phase retrieval, the sample was placed at four different distances behind the virtual source, and images were recorded by the CCD-based FReLoN detector. The four recorded intensity images contain complementary information about the X-ray phase at the sample position. Phase retrieval was then performed on the four images with the contrast transfer function method [4] to get the X-ray phase modulation due to the sample. The phase is proportional to the projection of the decrement of the refractive index, which in turn is given by the electron density. Propagation based phase imaging with a diverging beam is similar to the more familiar parallel beam case [5]. However the images are magnified by factor M = (z_1_+z_2_)/z_1_, and the propagation distance is replaced by D =z_1_z_2_/(z_1_+z_2_). Here z_1_ is the X-ray focus to sample distance and z_2_ is the sample to detector distance. The distances were chosen to get magnification that gave the desired pixel size of 60 nm, and to optimize the phase contrast in the images. It should be noted that the flat geometry of the specimen avoids strong refraction effects at the object boundaries that lead to artifacts in high resolution phase imaging. The lateral field of view was 90 × 90 μm^2^ at the largest magnification. 3D laminography reconstruction from typically 1200 to 2000 projections yields the electron density distribution. It was performed using a filtered back projection algorithm adapted to the laminography geometry [2]. The thickness of the sample that could be imaged is approximately equal to the lateral field of view.

Elemental composition imaging was done with the sample at the X-ray focus. A silicon drift diode detector was used for detecting the energy of the emitted fluorescence photons. The detector was placed in the horizontal plane at 90 degrees from the incident beam direction. This detector placement minimized the signal from scattered photons, because the horizontally polarized X-ray beam does not scatter elastically to the direction of the detector. This was an important design choice, because for trace element analysis the signal from the fluorescence is weak, and therefore high incoming intensity has to be used, to get sufficient count rates for practical imaging. If the scattering had been too strong, it would have degraded the detection limit by saturating the fluorescence detector and increasing the background. In order to minimize the path length of the emitted fluorescence photons inside the specimen, a horizontal rotation axis was chosen as shown in Figure 1A and Figure 3C. Therefore the self-absorption problem is reduced and its effect is uniform in any plane parallel to the specimen surface.

The specimen was raster scanned in the focus to get a 2D fluorescence map using a fast scanning procedure. For fluorescence laminography, such 2D scans were repeated for 64 rotation angles. Each projection contained 100 × 100 points with step size of 0.5 µm. For each point in the scan, a full spectrum with 2048 channels was stored. The fluorescence spectra were then processed by the software PyMCA [6] developed at the ESRF to get distributions of each element. Considering the limited number of recorded projections, an algebraic reconstruction technique [7] was used for 3D reconstruction.

For 3D image reconstruction, two important parameters are the exact laminographic angle and lateral location of the rotation axis. The laminographic angle was determined using a separate measurement with a spherical test object. The test object was placed off the rotation axis, and images were recorded by a laminographic scan. The test object follows an ellipsoidal trajectory and we determined the principal axis of the ellipse to get an accurate value for the laminographic angle. The lateral position of the rotation axis was determined for each specimen separately by summing up the projection images. Sample features trace ellipses in these images which were used to determine the lateral position of the rotation axis.

**Animal preparation protocol.** Male Sprague Dawley rats weighing 180-220 g were purchased from Charles River Laboratories (St Germain-sur-l’Arbresle, France). The rats were kept in a conventional animal facility and housed in positive-pressure air-conditioned units (22°C, 60% relative humidity) on a 12:12 h light/dark cycle. The experimental protocol has been approved by the local ethical committee for animals in research.

SWCNT used (CNI® Carbon nanotubes, product number R0560) was purchased from CNI (USA). These nanotubes were synthesized by Chemical Vapor Deposition method. Their diameter ranged from 0.8 to 1.2 nm, and their length from 0.1 to 1 µm. This SWCNT contained 10.1% Fe as catalyst (data obtained by INERIS ICP-OES dosage).

Groups of 6 rats were anesthetized (0.5 mg/kg ketamine hydrochloride, 0.1 mg/kg atropine and 1 mg/kg xylazine) and were intratracheally instilled with SWCNT dispersed with bovine serum albumin (BSA) as previously described [8] at a dose of 200 µg/rat in 150 µl. 7 days after the exposure, rats were sacrificed. Tissue specimens from the lung were fixed in 10% formaldehyde and processed routinely for embedding in paraffin. Slices of 40 µm thickness and 3×3 mm^2^ cross section were cut from the paraffin block sections and were used to perform 3D imaging.

**References:**

1. Ziedses des Plantes B-G (1932) Eine neue Methode zur Differenzierung in der Roentgenographie (Planigraphie). *Acta Radiol* 13: 182–192.
2. Helfen L, Myagotin A, Mikulík P, Pernot P, Voropaev A, et al. (2011) On the implementation of computed laminography using synchrotron radiation. *Rev Sci Instrum* 82: 063702.
3. Salvo L, Cloetens P, Maire E, Zabler S, Blandin J, et al. (2003) X-ray micro-tomography an attractive characterisation technique in materials science. *Nucl Instrum Methods Phys Res B* 200:273–286.
4. Cloetens P, Ludwig W, Baruchel J, van Dyck D, van Landuyt J, et al. (1999) Holotomography: Quantitative phase tomography with micrometer resolution using hard synchrotron radiation X-rays. *Appl Phys Lett* 75: 2912.
5. Mokso R, Cloetens P, Maire E, Ludwig W, Buffiere JY (2007) Nanoscale zoom tomography with hard X-rays using Kirkpatrick-Baez optics. *Appl Phys Lett* 90: 144104.
6. Solé VA, Papillon W, Cotte M, Walter P, Susini J (2007) A multiplatform code for the analysis of energy-dispersive X-ray fluorescence spectra. *Spectrochim Acta Part B At Spectrosc* 62: 63–68.
7. Kak AC, Slaney M (2001) *Principles of Computerized Tomographic Imaging* (SIAM, Philadelphia), pp 275–296.
8. Elgrabli D, Abella-Gallart S, Aguerre-Chariol O, Robidel F, Rogerieux F, et al. (2007) Effect of BSA on carbon nanotube dispersion for in vivo and in vitro studies. *Nanotoxicology* 1(4): 266–278.
